# Patterning of Nanocrystalline Cellulose Gel Phase by Electrodissolution of a Metallic Electrode

**DOI:** 10.1371/journal.pone.0099202

**Published:** 2014-06-04

**Authors:** Jean-Michel Daignan, Ran Chen, Khaled A. Mahmoud, Yuan Ma, Ian G. Hill, Laurent Kreplak

**Affiliations:** 1 Department of Physics and Atmospheric Science, Dalhousie University, Halifax, NS, Canada; 2 Department of Electrical and Computer Engineering, Dalhousie University, Halifax, NS, Canada; 3 Qatar Environment and Energy Research Institute (QEERI), Qatar Foundation, Doha, Qatar; Northeastern University, United States of America

## Abstract

At high concentration or in the presence of electrolytes and organic solvents, solutions of cellulose nanocrystals (CNCs) can form gels exhibiting optical properties similar to the ones of liquid crystal phases. In an attempt to pattern such a gel phase, we have studied the electrodissolution of a metallic electrode in a water suspension of carboxylated CNCs (cCNCs). Depending on the metal used, the electrodissolution process was observed at a different positive potential. In the case of copper the minimum potential at which we could observe optically the growth of the gel phase was 200 mV. The growth rate was current limited indicating that the process was controlled by the electrodissolution of the copper electrode. This hypothesis was confirmed by using circular and square copper patterns as positive electrodes. In both cases, the consumption of the electrode material was observed optically and correlated with the growth of the gel phase.

## Introduction

Cellulose nanocrystals (CNCs), isolated from cellulose fibers,[1], [2] have emerged as new class of nanomaterials. Robust cellulose nanocrystals (CNCs) with biocompatibility, high crystallinity and exceptional mechanical properties have been fostered in a myriad of applications including enzyme immobilization,[3] drug delivery,[4] and biomedical applications.[5] The most common process for the isolation of CNCs from cellulose fibers is based on controlled acid hydrolysis by removing amorphous regions of cellulose and leaving behind rod-shaped crystals. The aqueous dispersion produced by this method is electrostatically stabilized by formation of sulfate ester groups at the surface of the CNCs.[6] Recently, a new versatile one-step method has utilized ammonium persulfate, as an alternative to the more common acid process, to produce highly crystalline carboxylated CNCs.[7] The presence of surface carboxylic acid groups provides CNCs with active sites for facile surface modification and versatile applications.[8]

It was first reported by Marchessault and coworkers[9] that colloidal suspensions of cellulose nanocrystals produced by acid hydrolysis exhibit liquid crystalline properties. It was later demonstrated that CNCs can form a chiral nematic liquid crystal phase where the nanocrystals adopt a lamellar structure with a small angular mismatch between molecular layers giving rise to a long-range helical order [10], with unique optical properties, similar to the one observed for small molecule mesogens.[11] The chiral nematic liquid crystalline phase is lyotropic and the onset of the transition from isotropic to liquid crystalline occurs around 1 wt% depending on the source of CNCs. At high CNCs concentration around 10wt% the liquid crystalline phase dominates and the suspension turns to a gel. Increasing the ionic strength of a CNCs suspension can also result in phase separation into isotropic and gel phase.[12]–[14] In this context, aqueous suspensions of carboxylated CNCs (cCNCs) displayed birefringence patterns but did not flocculate or sediment owing to the polyanionic character imparted by the negative charges on the cCNCs surface.[15] Finally, the liquid crystalline phase was even observed in suspensions of CNCs with surfactants in an apolar solvent like cyclohexane where it exhibited a chiral nematic pitch as low as 2 µm.[16]

Beyond the propensity of CNCs to form a chiral nematic phase, they can also be aligned using both electric and magnetic fields.[17], [18] This alignment can be induced in both the isotropic and chiral nematic phase. In this study we take advantage of the alignment induced by an electric field and the gelation induced by ions to pattern the gel phase of cCNCs via the electrodissolution of a metallic electrode in an isotropic cCNC suspension.

## Materials and Methods

### Materials

Avicel microcrystalline cellulose and ammonium persulfate were obtained from Sigma-Aldrich. Metal wires of copper, silver, iron and gold, more than 99.9% pure and 100 µm in diameter, were purchased from Alfa Acer (Ward Hill, MA). Bundles of silver plated copper wires 200 µm in diameter were purchased from Omega (Stamford, CT).

### Preparation and characterization of carboxylated CNCs

As previously described,[7] Avicel microcrystalline cellulose (10 g) was suspended in one litre of 1 M ammonium persulfate. The suspension was heated to 60°C with vigorous stirring for 16 h. The resulting white suspension was purified from salts by repeated centrifugation/washing cycles (12,000 rpm for 10 min.) until the conductivity of the solution reached ∼5 µS·cm^−1^ (pH ∼6–7). The next step was to resuspend the product in double distilled water and spray dry or lyophilize to yield a white powder of cCNCs. Alternatively, we obtained CNCs samples prepared in a similar way from Biovision Technology (Nova Scotia, Canada). The dried CNCs powder was solubilized in water to a final concentration of 5.5wt% using sonication. The obtained suspension was isotropic as checked by polarized light microscopy (Figure S1, panel A). The zeta potential of a suspension of CNCs diluted to 2.75wt% in water was −65 mV as estimated from a mobility of −5 µm.cm.V^−1^.s^−1^ measured using a Zetasizer Nano ZS (Malvern Instruments, UK). This is double the zeta potential estimated for CNCs obtained from sulfuric acid hydrolysis and suspended in water.[19] As expected, the 5.5wt% suspension was also sensitive to ions, the addition of 10 mM NaCl made the suspension turbid and birefringence was observed by polarized light microscopy (Figure S1, panel B).

### Electrodissolution set-ups

Two electrodissolution set-ups were used in this study. The first set-up consisted of two metal wires with their ends attached on a glass slide and partially immersed in 50 µl of suspension at CNCs concentrations of 0.5 or 5.5wt%. The glass slide was mounted on the rotating stage of a transmission polarized light microscope (model KLSM from Carl Zeiss Canada) equipped with a Canon EOS 450D digital camera. A constant voltage was applied between the two wires using a HP 6284A DC power supply and the current in the circuit was measured using a computer controlled BK precision 5491A multimeter. In order to estimate the growth rate of the birefringent phase, we used ImageJ (http://rsb.info.nih.gov/ij/) to measure the radius of the growing phase on each frame acquired with the digital camera. The second set-up was identical except that one of the bundles was replaced 100 nm thick copper pads of circular or square shape deposited on ITO coated glass using an electron beam evaporator. The disks had a 250 µm radius and the squares had 1 mm sides. The deposition rate was 0.2 nm/s and the chamber pressure was 3 10^−6^Torr.

### Finite element modeling

The numerical simulations were performed using a commercial Finite Element Analysis (FEA) software – Ansoft Maxwell 2D/3D. The model consisted of a pair of 200 µm diameter copper electrodes immersed in pure water and separated by 500 µm. The volume of the water container was 50×20×20 mm^3^. The potential across the two electrodes was 1 V. The relative permittivity of water was set to 81, the bulk conductivity of water set to 0.01 S.m^−1^, and the bulk conductivity of copper was set to 58.10^6^ S.m^−1^.

## Results

**Figure 1:**
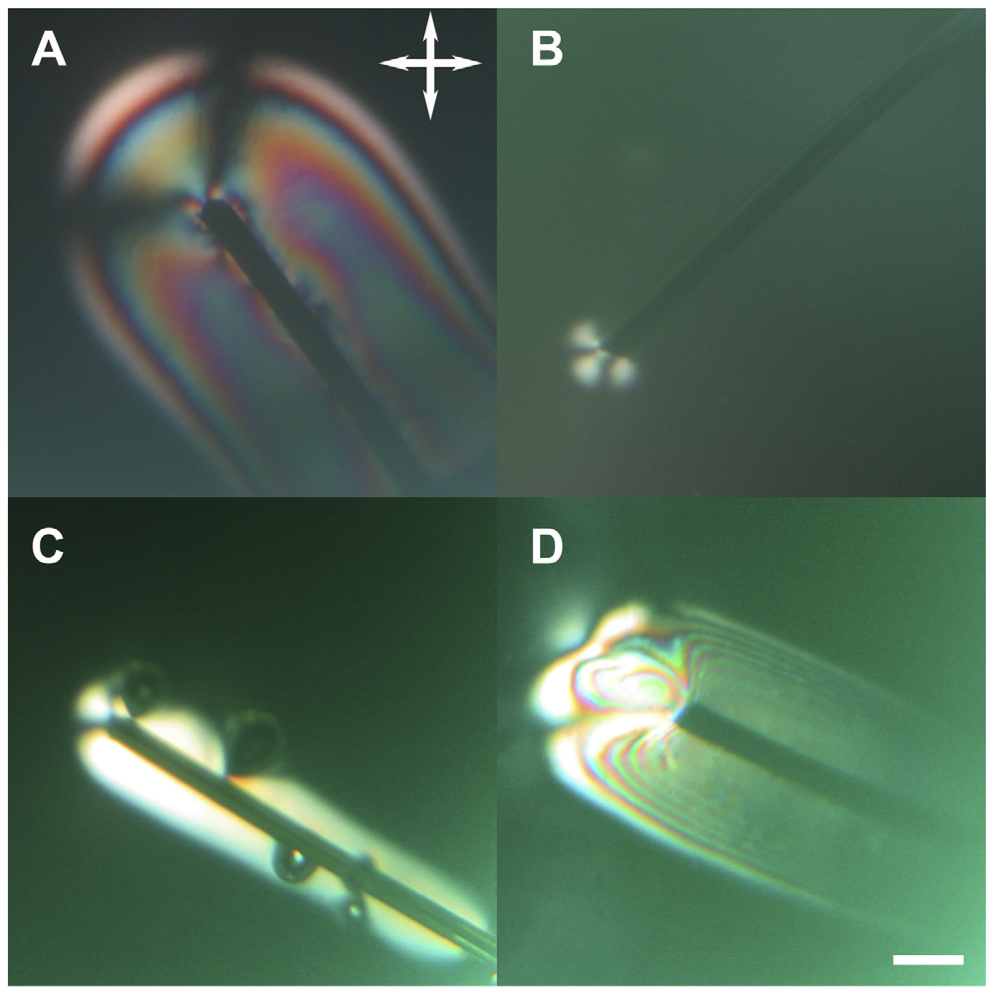
Optical images with crossed polarizers of four different metal wires immersed in a drop of 5.5wt% CNCs in water. Each wire was held at a constant positive potential with respect to a bundle of silver plated wires not shown in the field of view. A) Copper wire held at 1 V for 10 min. B) Iron wire held at 1 V for 7 min. C) Gold wire held at 2 V for 1 min. D) Silver wire held at 5 V for 30 s. The position of the crossed polarizers is drawn in panel A. The scale bar is 300 µm.

**Figure 2:**
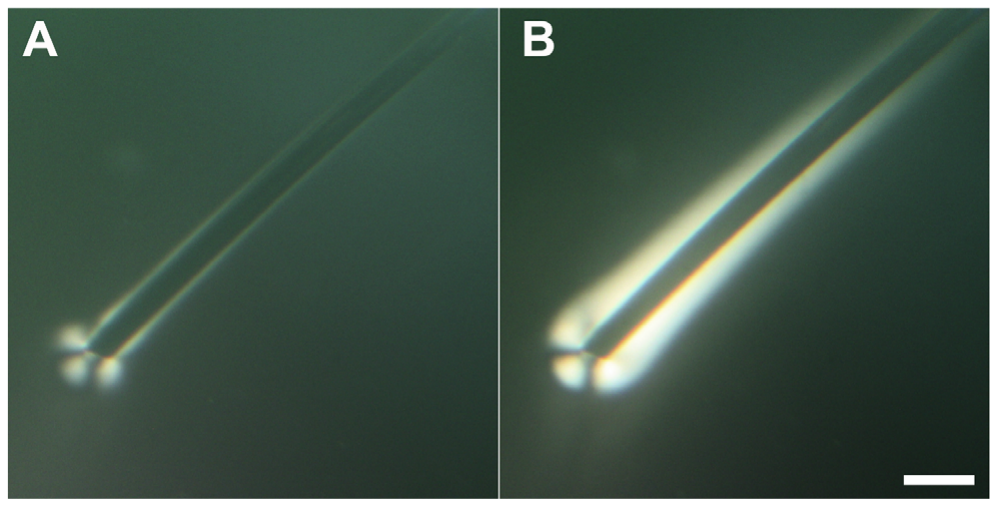
Optical images with crossed polarizers of an iron wire held first at 1(Figure 1B) and then held at 2 V. A) 30 s after the potential was increased to 2 V. B) 90 s after the potential was increased to 2 V. The position of the crossed polarizers is drawn in Figure 1 panel A. The scale bar is 300 µm.

In a first series of experiments, we applied a constant positive potential to a wire of a given metal with respect to a bundle of silver plated copper wires. Both electrodes were immersed in a drop of 5.5wt% CNCs in water. For the four metals used, copper, iron, gold and silver, we observed the growth of a gel phase around the wire under crossed polarizers (Figure 1), the experiment was repeated twice for each metal. The growth occurred only on the wire held at a positive potential as expected from the negative zeta potential of the CNCs in water. However, the threshold potential at which the gel phase was observed appeared to be metal dependent. It was below 1 V for copper and iron, and around 2 V for gold and silver. We also observed that the growth velocity seemed to increase qualitatively with applied potential (Figure 1, panels A and B versus panels C and D). In the case of the iron wire the growth was limited to the end of the wire as long as the potential was kept to 1 V. As soon as we increased the potential to 2 V the gel phase started to grow on the sides of the wire as well (Figure 2). This two-stage behavior disappeared when the sides of the wire were polished with sand paper just before starting the experiment (data not shown).

**Figure 3:**
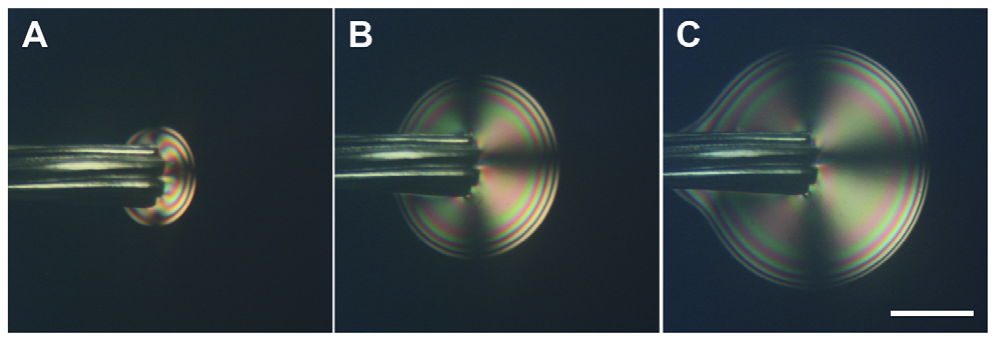
Optical images with crossed polarizers of a bundle of silver plated copper wires immersed in a drop of 5.5wt% CNCs in water and held at 1 V for A) 1 min, B) 5 min, C) 10 min. The position of the crossed polarizers is drawn in Figure 1 panel A. The scale bar is 300 µm.

In a second series of experiments, we decided to use two bundles of silver plated copper wires as electrodes and to keep the potential below 2 V. With this set-up, we only observed the gel phase growing at the end of the wire where the copper was exposed to the CNCs suspension (n = 20). The growth appeared concentric and centered in the middle of the exposed copper cross-section (Figure 3). After 5 min a ring structure was distinguishable at the edge of the gel phase and a well-defined maltese cross pattern was visible (Figure 3B). The ring structure is possibly an interference pattern due to the curvature at the surface of the gel and/or the signature of a lamellar structure. The maltese cross pattern is generally associated with a radial orientation of the molecules in for example nematic drops[20] indicating that the CNCs may be arranged in a radial pattern with respect to the copper cross-section. Interestingly the obtained gel phase did not dissociate after turning off the potential and gels made using this approach are stable for at least a year.

**Figure 4:**
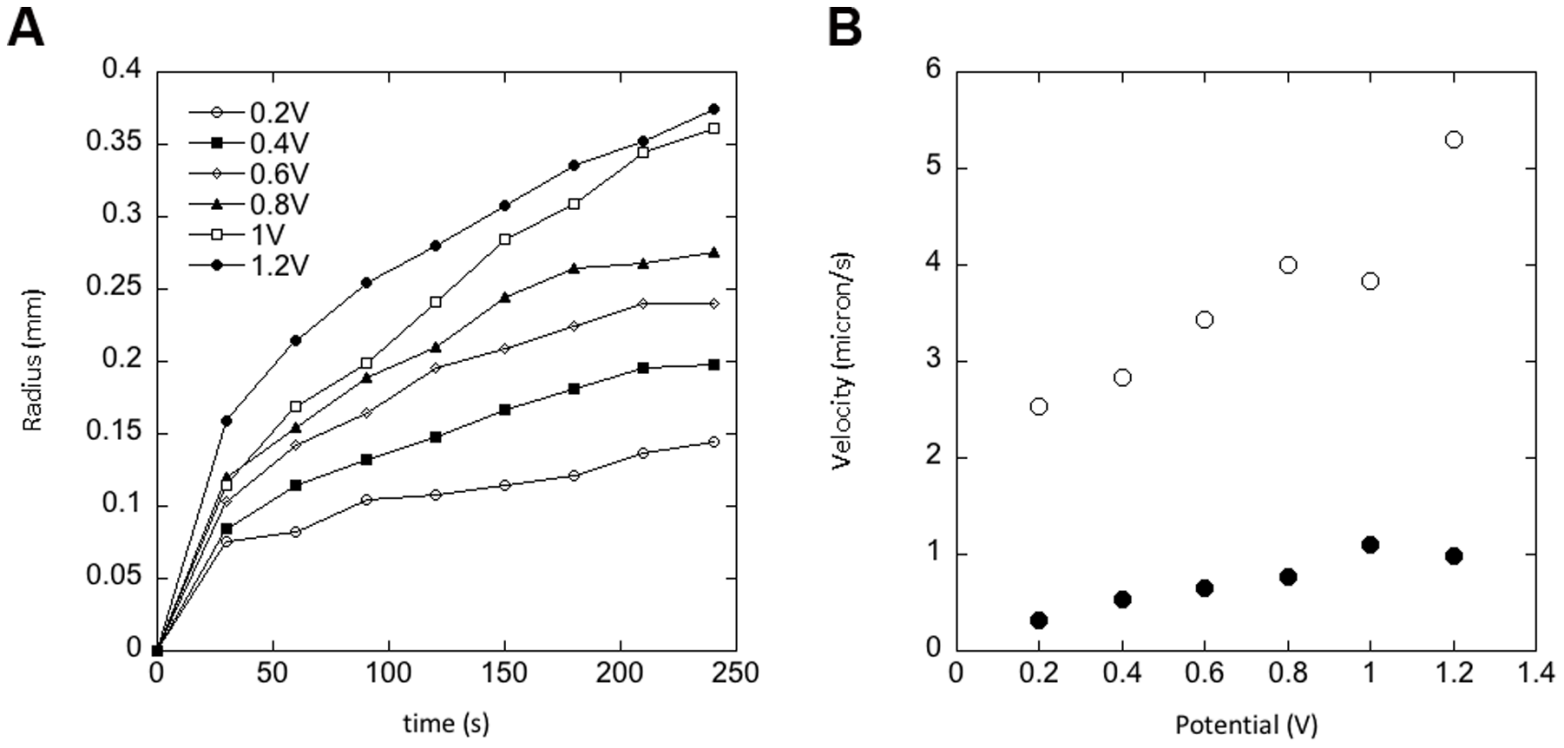
Growth of the gel phase around a bundle of silver plated copper wires immersed in a drop of 5.5wt% CNCs in water and held at different potentials from 0.2 to 1.2 V. A) radius of the gel phase as a function of time for each applied potential. B) Average growth velocity as a function of applied potential for the first 30 s (solid symbols) and after (open symbols).

**Figure 5:**
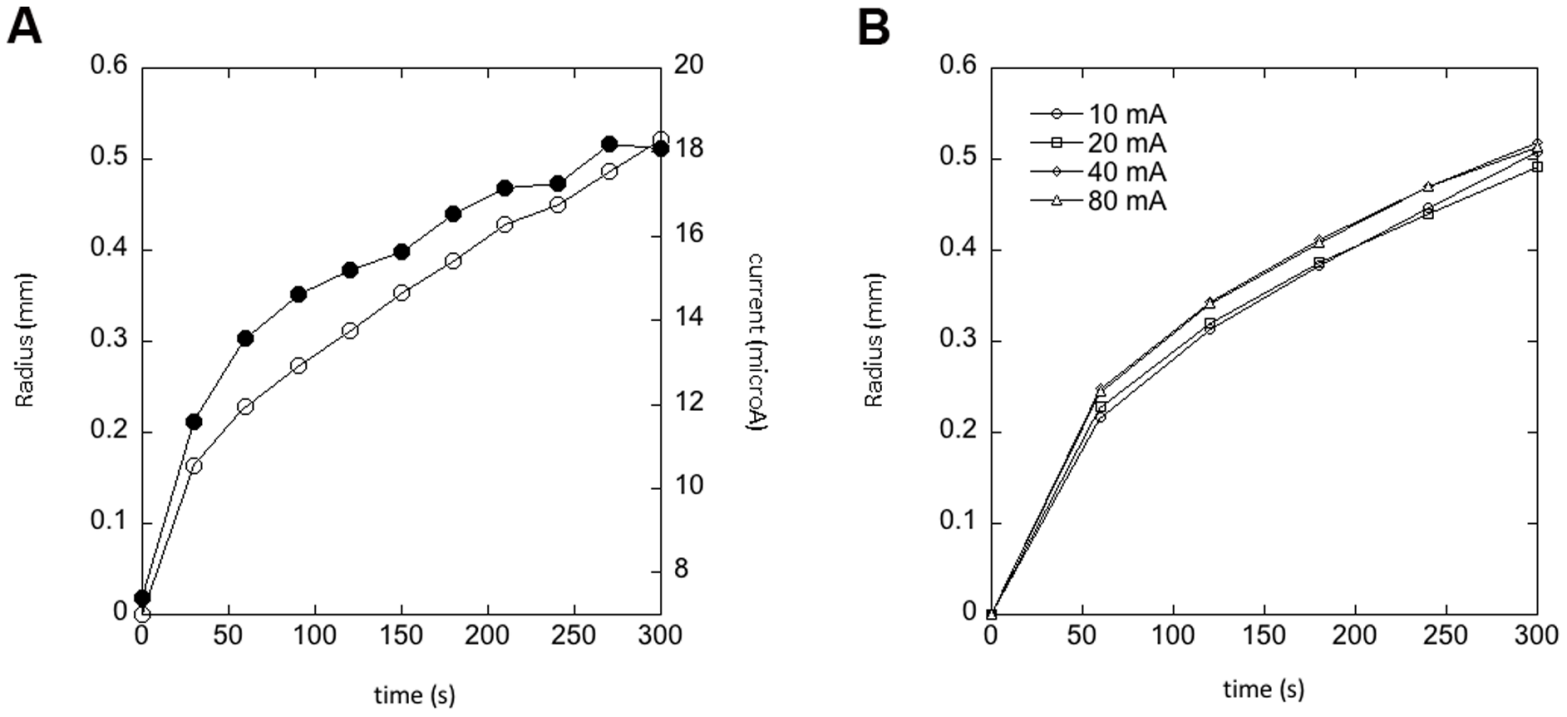
Growth of the gel phase around a bundle of silver platted copper wires immersed in a drop of 5.5wt% CNCs in water and held at 1 V. A) Radius of the gel phase (open symbols) and current between the two bundles (solid symbols) as a function of time. B) Radius of the gel phase as a function of time for different current forced between the two bundles.

In order to understand the effect of the applied potential, we followed the growth of the gel phase by recording the radius of the phase with respect to the center of the bundle cross-section, for different potentials from 0.2 to 1.2 V. Below 0.2 V we did not observe a gel phase. We observed that the growth occurred in two distinct regimes that were both dependent on the applied potential (Figure 4). Over the first 30 s the radius increased at an average velocity of 2 to 5 µm/s and then the velocity dropped abruptly to 0.3 to 1 µm/s (Figure 4A). In both regimes the velocity increased linearly with the applied potential (Figure 4B). In a separate experiment we recorded the current in the circuit as a function of time during the growth of the gel phase for an applied potential of 1 V (Figure 5A). We observed a current in the µA range that behaved similarly than the radius of the gel phase (Figure 5A). In order to test if the growth was current limited we used a current source to apply a 1 V potential and force 10 to 80 mA of current into the circuit. The radius of the gel phase behaved as shown in Figure 5A with two growth regimes but the growth velocities appeared weakly dependent on the current in that range (Figure 5B).

**Figure 6:**
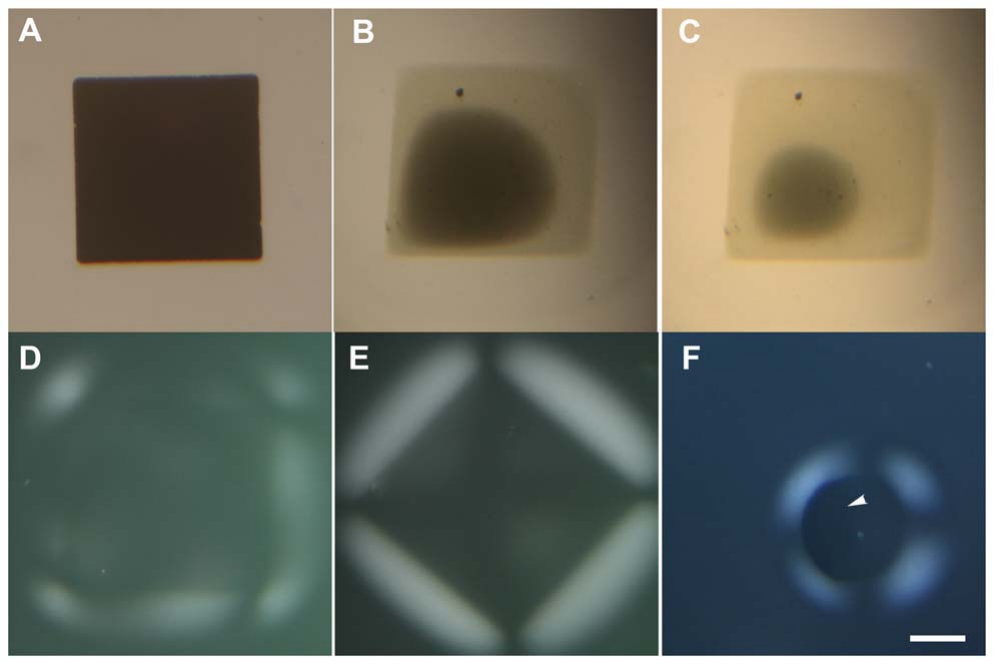
Growth of the gel phase around copper pads on a ITO substrate immersed in a drop of 0.5wt% CNCs in water and held at 1 V. A) square of copper before the experiment. B) Square of copper after 27 min. C) square of copper after 28 min. D) square of copper after 32 min imaged with crossed polarizers. E) Same as D) but the square was rotated by 45°. F) disk of copper after 7 min, the copper is still visible on the left side of the disk, arrowhead. The position of the crossed polarizers is drawn in Figure 1 panel A. The scale bar is 300 µm.

Considering that the growth of the gel phase necessitated a minimum applied potential and that it seemed to match the ionic current between the two electrodes, we hypothesized that the appearance of a gel phase was associated with the removal of positive ions from the electrode surface. In order to test this hypothesis we fabricated 100 nm thick squares and disks of copper onto ITO coated glass. We applied a 1 V potential between the ITO and a wire bundle immersed in a drop of 0.5% CNCs placed above one of the copper pads (Figure 6). For both types of pads we observed the disappearance of the copper over time (Figure 6B, C and F) and the growth of the gel phase around the pad (Figure 6D, E and F). Interestingly the optical images of the gel phases grown using this set-up were very reproducible (n = 3 for each type) and seemed to only depend on the geometry of the copper pads and the orientation of the pads with respect to the crossed polarizers (compare Figure 6D and E).

## Discussion

**Figure 7:**
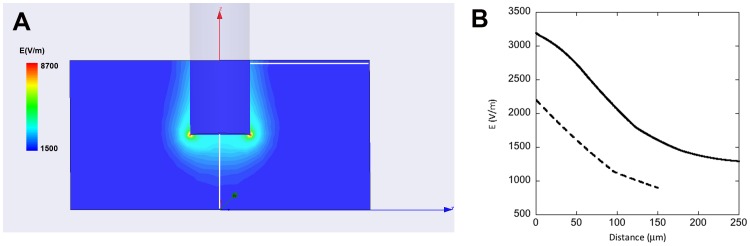
3D finite element model of the cylindrical electrode geometry used in this study performed in pure water and for an applied potential of 1 V. A) equipotentials around a cylindrical electrode in a plane going through the centre of the cylinder. B) Electric field as a function of distance from the electrode surface taken along the two white lines in A). The solid line corresponds to the white line at the end of the electrode and the broken line corresponds to the white line perpendicular to the side of the electrode.

It is already well known that solutions of CNCs in water can form a gel phase in the presence of ions.[12]–[14] In this study we demonstrated that the electrodissolution of a metallic electrode controlled the growth of such a gel phase. The electrodissolution of metals, and especially copper, has been mainly studied in the presence of acid solutions.[21], [22] However it is clear from this study that electrodissolution can occur in water, in the presence of few ions, provided a high enough potential is applied. We suspect that this is due to an acidification of the solution surrounding the positive electrode as demonstrated for two bundles of silver plated copper wires immersed in a 0.03% phenol red solution (Figure S2). In principle any metal could be used even so we have only demonstrated the effect for four elements, copper, iron, silver and gold (Figure 1). The growth of the gel phase itself is controlled to some extent by the ionic current between the two electrodes (Figure 5A). Still, there is a maximum ionic current that can be used to sustain the growth. Forcing more currents into the circuit beyond that point does not accelerate the process further (Figure 5B). Overall it is possible to achieve temporal control of the growth process by tuning the amount of current between the two electrodes. Another interesting aspect is spatial control. Since the threshold potential for electrodissolution is metal dependent (Figure 1), it is possible to spatially control the growth of the gel phase using two metals with different threshold potential such as copper and silver (Figure 3). Still this does not explain the overall shape of the gel and its appearance when observed under crossed polarizers (Figure 3 and 6). Considering that CNCs can be aligned under an electric field [18], we used finite element modeling to calculate the strength and shape of equipotential lines around a wire immersed in water (Figure 7). The calculated equipotential surfaces are concentric cylinders along the wire side and mostly hemispherical at the end of the wire (Figure 7A). The field strength is the strongest along the rim of the wire end and it is overall much larger at the end of the wire compared to its side (Figure 7B). This provides a simple interpretation of our experiment with a iron wire in which we induced the growth of the gel phase first at the end of the wire and then along its side by increasing the applied potential from 1 V to 2 V (Figure 2). At 1 V, we propose that the effective potential was below the threshold for breaking the oxide layer along the wire side and above the threshold at the wire end. Increasing the potential to 2 V allowed the growth to occur everywhere along the wire as for the other metals (Figure 1). This interpretation was confirmed by mechanically polishing the sides of the wire that allowed the growth to occur everywhere on the wire at the same potential (data not shown). The fact that the equipotential surfaces are spherical at the wire end is also in good agreement with what we observed with the bundle of silver platted copper wires (Figure 3). Based on the optical images and the simulations it is reasonable to propose that the CNCs are either oriented parallel or perpendicular to the equipotential surfaces that we calculated. In the case of the gel phase obtained with the silver wire, we observed layers of alternating color along the wire side (Figure 1D) and we observed layers around 500 nm thick on the same dried sample using scanning electron microscopy (Figure S3). Based on this result and the electric-field simulations it is likely that the gels obtained in this study are layered structures reminiscent of the chiral nematic phase observed at high CNC concentration by other groups.[10]

## Conclusion

We demonstrated that it is possible to control the growth and possibly the morphology of a CNC gel phase using the electrodissolution of a metallic electrode. This simple technique opens the road for the patterning of a CNC gel using conventional photolithography techniques. We also provided preliminary evidence that these gels are layered arrangements of CNCs that resemble the typical architecture of a chiral nematic phase.

## Supporting Information

Figure S1 **Effect of ions on the birefringence of a concentrated CNC solution in water.**
(DOCX)

Figure S2 **pH change around the two metallic electrodes when 1V is applied.**
(DOCX)

Figure S3 **Scanning electron microscopy images of a dried CNC deposit obtained by electrodissolution of a silver electrode.**
(DOCX)
