# Supplementary material for: Patterning of Nanocrystalline Cellulose Gel Phase by Electrodissolution of a Metallic Electrode
Source: PLoS One. 2014 Jun 4;9(6):e99202. doi: 10.1371/journal.pone.0099202 (PMC4045955; doi:10.1371/journal.pone.0099202)
Supplement: Figure S1 — Effect of ions on the birefringence of a concentrated CNC solution in water. (DOCX) [file pone.0099202.s001.docx]

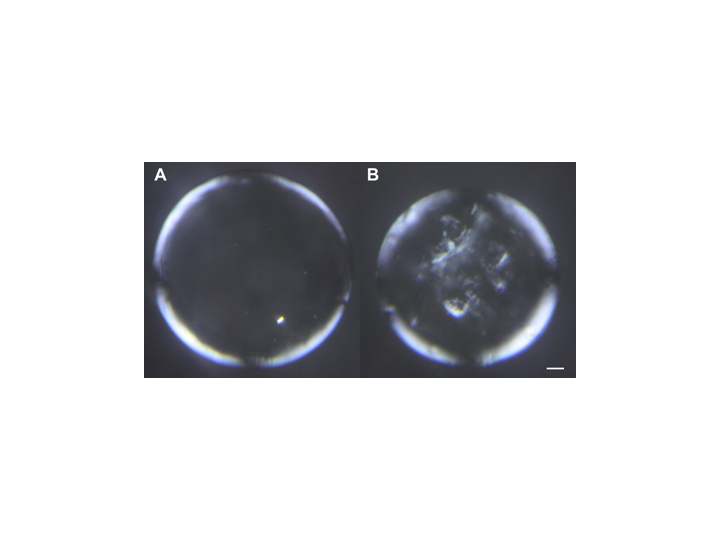


Figure S1: Optical images with crossed polarizers of two drops of 5.5wt% CNCs in water (A) and 5wt% CNCs in 10 mM NaCl (B). Some birefringence is always visible along the rim due to the alignment of CNCs upon drying. Scale bar 250 μm.
