# Supplementary material for: Patterning of Nanocrystalline Cellulose Gel Phase by Electrodissolution of a Metallic Electrode
Source: PLoS One. 2014 Jun 4;9(6):e99202. doi: 10.1371/journal.pone.0099202 (PMC4045955; doi:10.1371/journal.pone.0099202)
Supplement: Figure S2 — pH change around the two metallic electrodes when 1V is applied. (DOCX) [file pone.0099202.s002.docx]

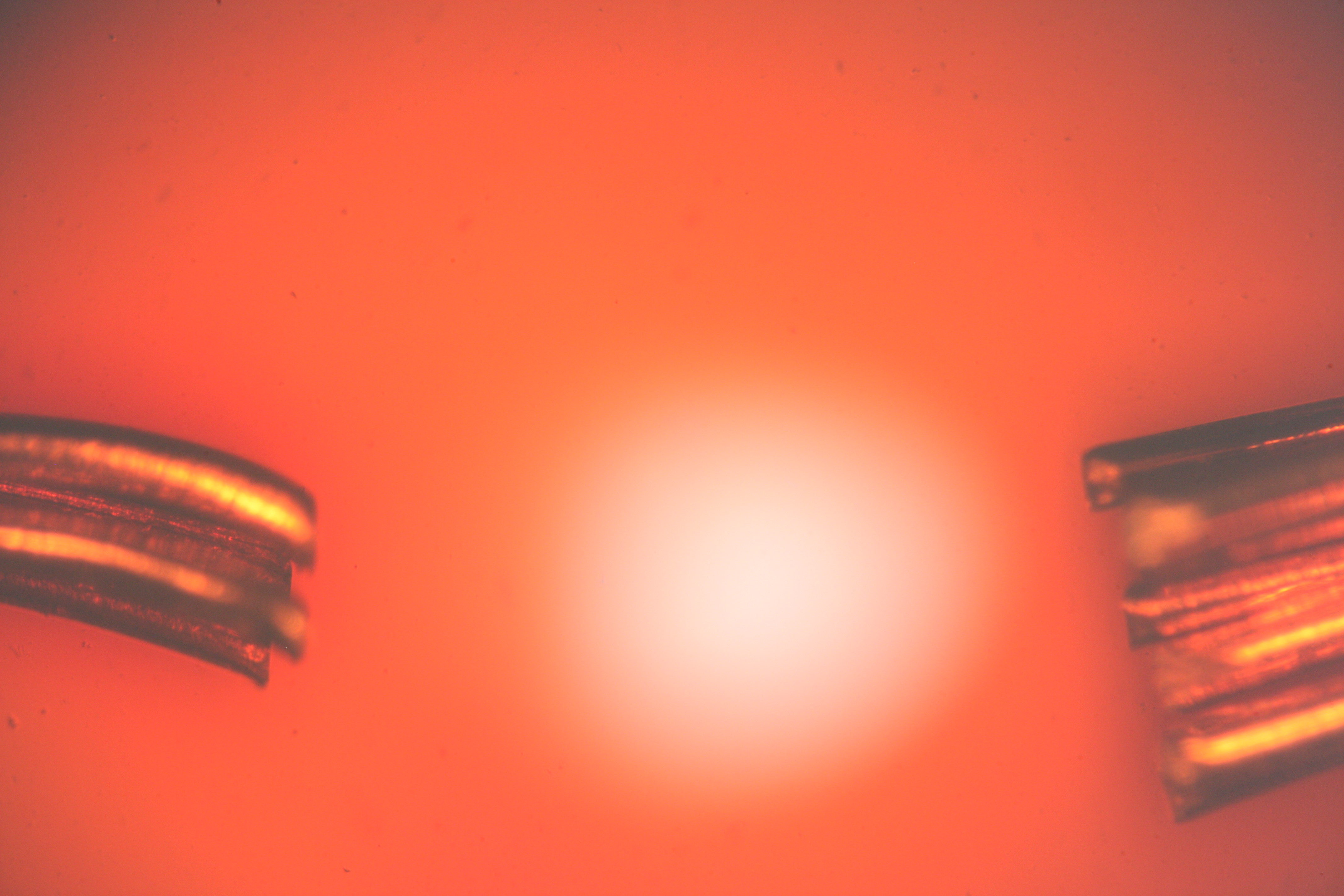

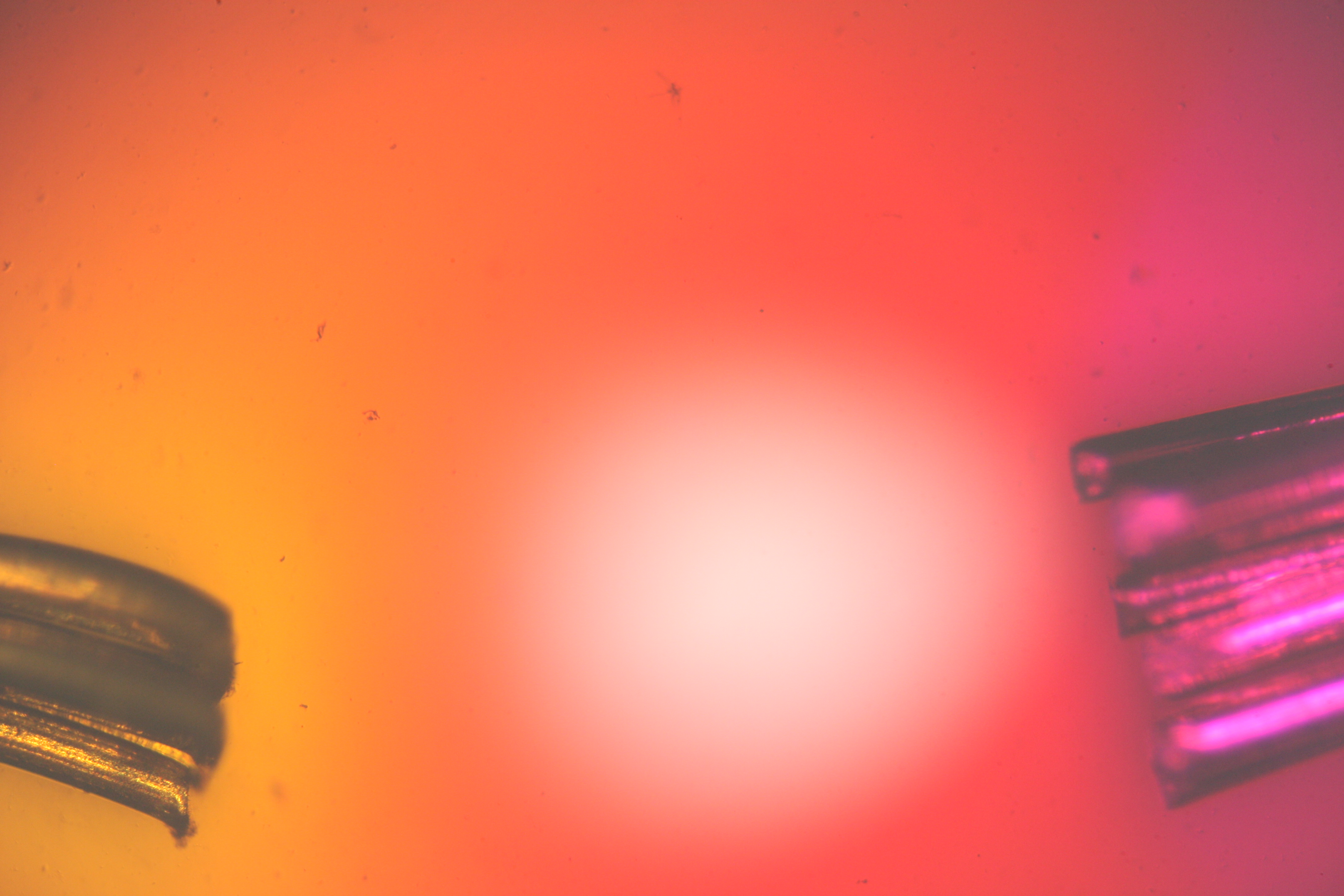


**A**

**B**

**+**

**+**

**-**

**-**

Figure S2: Brightfield optical images of two bundles of silver plated copper wires immersed in a drop of 0.03% phenol red in water and held at 1V for A) 0 min and B) 4 min. The large bright spot at the center of both images is due to the reflection of the illumination beam at the surface of the drop. Phenol red turns yellow for pH below 7 and purple for pH above 7. The yellow region surrounding the positive electrode in B) indicates acidification. Scale bar 500 μm.
