# Supplementary material for: Patterning of Nanocrystalline Cellulose Gel Phase by Electrodissolution of a Metallic Electrode
Source: PLoS One. 2014 Jun 4;9(6):e99202. doi: 10.1371/journal.pone.0099202 (PMC4045955; doi:10.1371/journal.pone.0099202)
Supplement: Figure S3 — Scanning electron microscopy images of a dried CNC deposit obtained by electrodissolution of a silver electrode. (DOCX) [file pone.0099202.s003.docx]

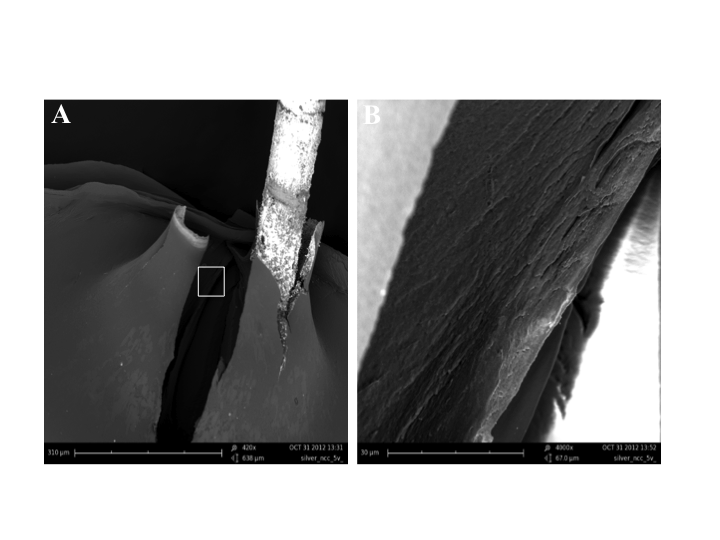


Figure S3: Scanning electron microscopy (SEM) images of the dried CNC liquid crystalline phase around the silver electrode in Figure 1D. A) overview image, the rectangular box corresponds to the region imaged in panel B. During the drying process the CNC structure delaminated from the silver wire giving us access to the silver/CNC interface. B) high magnification image of the silver/CNC interface showing a lamellar structure. Images were taken on a Phenom G2 SEM (Phenom, USA).
